# From the archives: regulation of mRNA translation, epigenetic machinery components, and processing of a composite large subunit ribosomal RNA

**DOI:** 10.1093/plcell/koad102

**Published:** 2023-04-10

**Authors:** Johan Zicola

**Affiliations:** Assistant Features Editor, The Plant Cell, American Society of Plant Biologists; Center for Integrated Breeding Research, Department of Crop Sciences, The University of Göttingen, 37073 Göttingen, Germany

## July 2022: regulation of mRNA translation

Glycine-rich RNA-binding proteins (GR-RBPs) are a superfamily involved in plant developmental and stress responses. GR-RBPs regulate several processes of RNA metabolism, including RNA editing, splicing, and transport (Ma et al. 2021). One year ago, Ma et al. (2022) explored the function of SlRBP1, the most expressed GR-RBP in tomato (*Solanum lycopersicum*) fruits. Transgenic knockdown *slrbp1* plants are dwarves with yellow fruits and leaves (see Figure). In addition, many genes involved in photosynthesis are misregulated in the mutant, suggesting a role of SlRBP1 in chloroplast function. Using co-immunoprecipitation followed by mass spectrometry of a tagged SlRPBP1 protein, the team found as an interactor the translation initiation factor SleIF4A2. Using RNA immunoprecipitation with antibodies against SleIF4A2, they showed that the SlRPBP1-SleIF4A2 complex binds to the transcripts of several photosynthetic genes. Furthermore, the transcripts of the target genes showed imbalanced polysome profiles in the *slrbp1* mutants, indicating that the SlRPBP1-SleIF4A2 complex promotes translation. This study shows that GR-RBPs in plants play a role in regulating translation.

**Figure.**
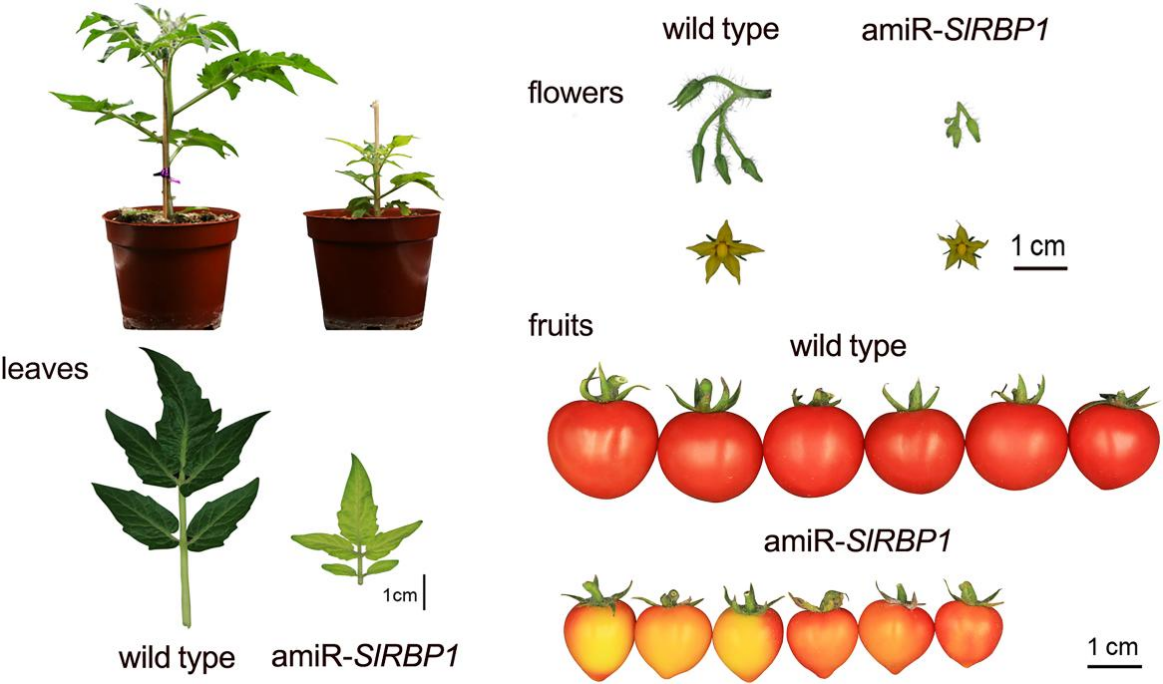
Knockdown of SlRBP1 affects vegetative and reproductive growth in tomato. Representative images of 5-week-old plants, leaves, flowers, and fruits of wild-type (*S. lycopersicum* cv. Micro-Tom) and amiR-SlRBP1 plants. Adapted from Ma et al. (2022), Figure 1.

## July 2018: epigenetic machinery components

In plants, epigenetic regulation is partly mediated by histone 3 lysine 27 trimethylation (H3K27me3), a repressive histone mark catalyzed by the polycomb repressive complex 2 (PRC2). Mateo-Bonmatí et al. (2018) characterized the role of *INCURVATA11* (*ICU11*) and *CUPULIFORMIS2* (*CP2*) in epigenetic regulation in Arabidopsis (*Arabidopsis thaliana*). The *icu11* mutant displays incurved leaves and early flowering, phenocopying the PRC2 mutant *curly leaf.* The group mapped the *icu11* mutation to a gene encoding a 2-oxolutarate/Fe(II)-dependent dioxygenase (2OGD). The team found that *ICU11* was not yet classified as a 2OGD, and based on sequence homology, they identified 4 additional paralogs they named *CP2* to *CP5.* They classified the 5 paralogs into a new 2OGD family named CUPULIFORMIS. They tested if *CP2* and *ICU11* have redundant functions because of their high sequence similarity. Although the *cp2* mutant resembles the wild type, the double mutant *icu11 cp2* shows severe developmental anomalies, indicating that *ICU11* and *CP2* are unequally redundant. To determine if *ICU11* is involved in PRC2 activity, the group looked at the expression of different flowering genes silenced by H3K27me3 and found several upregulated in the *icu11*. Upregulated genes also showed reduced H3K27me3 levels, supporting the role of *ICU11* in PRC2 function. A later publication showed that ICU11 physically associates with the PRC2 and probably demethylates the active histone mark H3K36me3 to allow H3K27me3 deposition by the PRC2 (Bloomer et al. 2020). Further work is needed to confirm the demethylase activity of ICU11 on H3K36me3.

## July 1998: processing of a composite large subunit rRNA

Ribosomes are composed of ribosomal RNAs (rRNAs) and proteins. In photosynthetic organisms, the chloroplasts encode their own rRNAs. In the alga *Chlamydomonas reinhardtii*, the 23S-like large subunit (LSU) contains a self-splicing intron. In the ribosome-deficient *ac20* mutant, the intron and the 3 internal transcribed spacers (ITSs) of the LSU are not properly removed, resulting in pre-rRNA accumulation and poor growth. Holloway and Herrin (1998) investigated the molecular basis of the *ac20* phenotype. Using Northern blot probes for different regions of the LSU in *ac20*, they detected the presence of pre-rRNAs at different processing stages, suggesting that the intron and the 3 ITSs are independently removed. They also generated transgenic algae in the *ac20* background with a deleted intron, but the *ac20* phenotype remained, indicating that the defect in intron splicing is not the primary cause of the *ac20* phenotype. To determine if the splicing of the intron is required for ITS processing, the team created transgenic lines with single-point mutations within the *I-CreI* homing endonuclease gene encoded in the intron. The only cells recovered contained mutations that did not entirely abolish intron splicing, and ITSs continued to be processed, indicating that intron splicing was not directly affecting ITS processing efficiency. The authors suggested a sequential processing pathway, where the intron is spliced first, consecutively followed by ITS-1, ITS-2, and ITS-3 removal.
